# Serum TNF-*α* Level Is Associated with Disease Severity in Adult Patients with Immunoglobulin A Vasculitis Nephritis

**DOI:** 10.1155/2020/5514145

**Published:** 2020-11-25

**Authors:** Haiting Wu, Yubing Wen, Cai Yue, Xuemei Li, Ruitong Gao

**Affiliations:** Division of Nephrology, Department of Internal Medicine, Peking Union Medical College Hospital, Chinese Academy of Medical Sciences & Peking Union Medical College, Beijing 100730, China

## Abstract

**Background:**

Tumor necrosis factor-*α* (TNF-*α*) is a proinflammatory factor involved in the pathogenesis of immunoglobulin A vasculitis (IgAV). The association between serum TNF-*α* and disease severity in adult patients with IgAV nephritis (IgAV-N) has been inadequately evaluated.

**Methods:**

Serum TNF-*α* was measured by chemiluminescence immunoassay in 53 renal biopsy-proved IgAV-N patients, 53 healthy controls, and 53 IgA nephropathy (IgAN) patients. The correlations of clinicopathologic parameters of IgAV-N patients with serum TNF-*α* were analyzed.

**Results:**

In this cross-sectional study, the median age of IgAV-N patients was 29 (25-37) years, and 67.9% were female. Serum TNF-*α* was significantly higher in the IgAV-N group than in the healthy group [7.4 (5.7-9.4) pg/mL *vs.* 5.9 (5.0, 7.1) pg/mL, *P* = 0.001], but comparable with sex, age, and estimated glomerular filtration rate (eGFR) grade-matched IgAN patients. Serum creatinine (*P* = 0.006) and serum cystatin C (*P* = 0.001) were positively correlated with serum TNF-*α* level, while albumin (*P* = 0.014) and eGFR (*P* = 0.021) were negatively correlated with serum TNF-*α* level. Multivariate linear regression analysis revealed that eGFR (*P* = 0.007) was an independent clinical predictor of serum TNF-*α*. Patients with higher pathological classification grade also had higher serum TNF-*α*.

**Conclusions:**

Serum TNF-*α* is associated with renal function and the pathological classification of adult patients with IgAV-N. TNF-*α* is a potential biomarker for the assessment of IgAV-N severity.

## 1. Introduction

Immunoglobulin A vasculitis (IgAV) is a small vessel systemic vasculitis that primarily affects children especially those less than 10 years old. Renal involvement is observed in approximately 30% of children, the severity of which highly impacts prognosis [1, 2]. In adults affected by IgAV, the incidence of renal involvement rises to more than 50%, and IgAV nephritis (IgAV-N) in adults is more severe with a worse prognosis [3]. The exact pathogenesis of IgAV-N has not been fully elucidated. It is known that the mucosal immune system is activated after antigen exposure, leading to the synthesis of hypogalactosylated IgA1 and anti-IgA1 antibodies. Aberrant IgA circulating immune complexes deposit in renal mesangial area, activating resident cells, complement system, and cytokines [2]. The proinflammatory cytokine, tumor necrosis factor-*α* (TNF-*α*), plays an important role in the inflammatory response in IgAV-N. TNF-*α* can regulate the mucosal immune response from multiple aspects [4, 5] and causes direct damage to renal tissue [6, 7]. Previous studies have shown that serum TNF-*α* is associated with disease severity in children [8–11]. However, the evaluation of serum TNF-*α* in adult patients is inadequate.

IgAV-N probably shares a common pathogenesis with IgA nephropathy (IgAN) as the two diseases have similar characteristics such as an abnormal IgA1 glycosylation pattern, deposition of mesangial IgA complexes, and mesangial proliferation. However, the pathophysiological mechanisms may be divergently different, leading to histological and clinical differences in the two diseases [2]. Our previous study showed that serum TNF-*α* was significantly correlated with renal function, urine protein excretion, and pathological classification of IgAN [12]. It is unknown whether serum TNF-*α* in IgAV-N shows a similar pattern to that in IgAN.

In the present cross-sectional study, we detected serum TNF-*α* levels in adult patients with IgAV-N, determined its role in severity assessment, and compared the serum TNF-*α* level in patients with IgAV-N and those with IgAN. TNF-*α* was measured by chemiluminescent immunoassay, a more sensitive, rapid, and stable quantitative method compared with the more commonly used enzyme-linked immunosorbent assay [13].

## 2. Subjects and Methods

### 2.1. Subjects

Serum and urine samples from 53 renal biopsy-proved IgAV-N patients aged 18 years or more were obtained from Peking Union Medical College Hospital (PUMCH) between August 2016 and September 2019. Patients with other renal diseases, systemic immune diseases, and malignancies were excluded. All the patients included had a history of skin purpura and renal manifestations. Renal biopsy showed typical features such as mesangial proliferation and IgA deposition in the mesangial area. Samples from 53 healthy controls and 53 IgAN patients matched by age and sex were also obtained from PUMCH. IgAN patients were also matched by and estimated glomerular filtration rate (eGFR) grade. This study was approved by the local Ethical Committee.

### 2.2. Measurement of Serum TNF-*α*

Blood samples from patients and controls were allowed to clot at room temperature for 30 min, centrifuged for 10 min at 3000 rpm, and the serum was immediately extracted to avoid the release of TNF-*α* from blood cells. Serum TNF-*α* was measured by chemiluminescent immunoassay using the IMMULITE® 1000 system (Siemens Healthcare Diagnostics Inc., United Kingdom) with the recommended reference range ≤ 8.1 pg/mL.

### 2.3. Clinical, Laboratory, and Histologic Data

Medical records were reviewed for demographic information, physical examination, and laboratory tests such as complete blood count, serum creatinine (sCr), serum cystatin C (Cys-C), hypersensitive C-creative protein (hsCRP), routine urinalysis, and 24-h urine protein excretion. eGFR was calculated by the Chronic Kidney Disease Epidemiology Collaboration (CKD-EPI) equation [14]. Renal function was graded as chronic kidney disease (CKD) stages 1–5 according to the Kidney Disease: Improving Global Outcomes classification [eGFR ≥ 90 (G1), 60–89 (G2), 30–59 (G3), 15–29 (G4), and <15 (G5) mL/min/1.73 m^2^] [15]. Renal pathology was classified as type I-V using the International Study of Kidney Disease in Children (ISKD) classification [3]. Treatments including renin-angiotensin system inhibitors (RASI), glucocorticoids, and immunosuppressants were recorded.

### 2.4. Statistics

Data were expressed as the median (interquartile range) or absolute numbers (frequency). The Mann–Whitney test, Kruskal-Wallis test, or Chi-square test was used to analyze differences between groups. Spearman's rank test was used to determine the correlations of continuous variables. TNF-*α* was adjusted by logarithmic transformation before multiple linear regression. The clinical indicators showing correlation in univariate analysis will be included in multiple linear regression with stepwise analysis. Statistical analysis was performed using SPSS statistical software version 23.0 (SPSS Inc., Chicago, IL, USA). *P* < 0.05 was considered statistically significant.

## 3. Results

### 3.1. Demographic and Clinical Features

**Figure 1:**
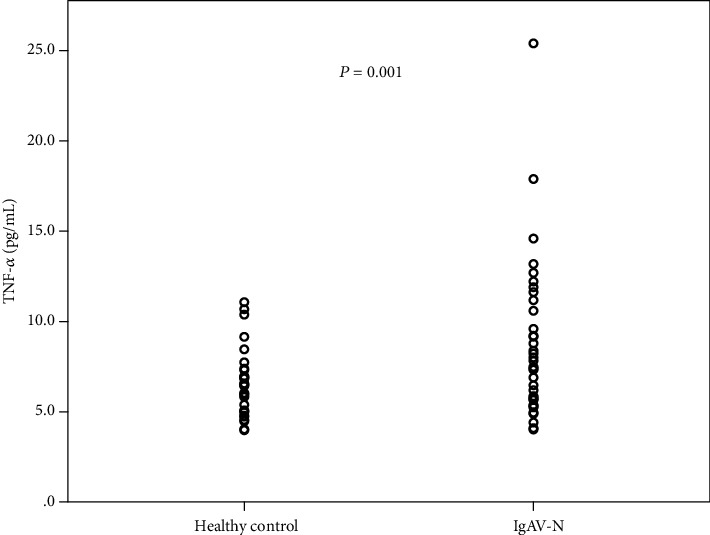
Serum tumor necrosis factor alpha (TNF-*α*) in healthy controls and IgA vasculitis patients with nephritis.

The median age of the 53 IgAV-N patients was 29 (25-37) years, and 67.9% were female. The median age of the 53 healthy controls was 30 (27-34) years, and 60.3% were female. Serum TNF-*α* was significantly higher in the IgAV-N group [7.4 (5.7-9.4) pg/mL *vs.* 5.9 (5.0-7.1) pg/mL, *P* = 0.001] (Figure 1). Most IgAV-N patients had well-preserved renal function, with 66.0% CKD stage 1 and 22.6% stage 2. Proteinuria was mild to moderate. And 58.5% of patients were treated with RASI, 39.6% with glucocorticoids, and 34.0% with immunosuppressants, of which mycophenolate mofetil was the most frequently administered drug, followed by cyclophosphamide and calcineurin inhibitors. Other demographic and clinical manifestations are shown in Table 1.

### 3.2. Correlation between TNF-*α* and Clinical Manifestations

Patients were divided into the high and low TNF-*α* groups according to the upper normal limit of TNF-*α* detection (8.1 pg/mL) (Table 1). The high TNF-*α* group had higher sCr (*P* = 0.049) and Cys-C (*P* = 0.036) levels. Although the average eGFR was not different between the groups, more patients in the high TNF-*α* group were CKD G3 and fewer were CKD G1 than in the low TNF-*α* group (*P* = 0.032). The high TNF-*α* group also had higher IgA levels (*P* = 0.004), while proteinuria and hematuria were comparable. More patients received glucocorticoid treatment in the high TNF-*α* group (*P* = 0.037). Correlation analysis showed that sCr (*P* = 0.006) and Cys-C (*P* = 0.001) were positively correlated with serum TNF-*α* level, while albumin (*P* = 0.014) and eGFR (*P* = 0.021) were negatively correlated with serum TNF-*α* level (Table 2). Multivariate regression analysis revealed that renal function (due to collinearity of sCr, eGFR, and Cys-C, only eGFR was included in the multiple linear regression analysis) was an independent predictive factor.

### 3.3. Pathological Classification and Correlation between Pathology and Serum TNF-*α*

**Figure 2:**
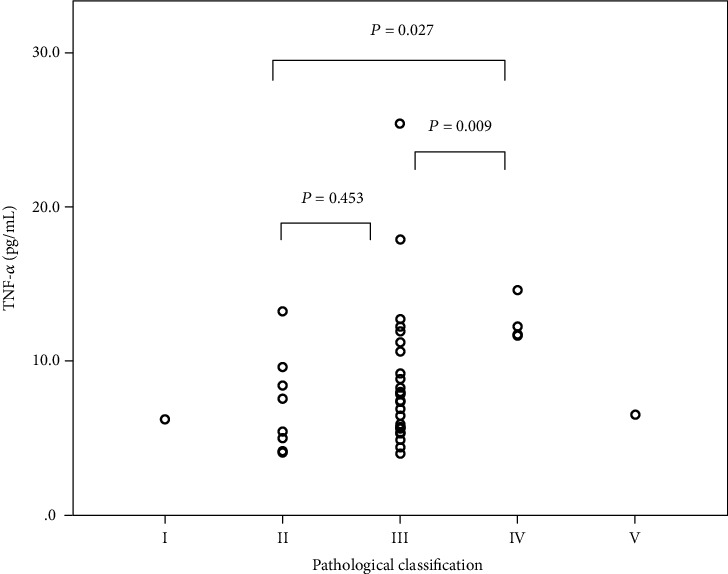
Serum TNF-*α* level in different renal pathological classes of IgA vasculitis. Patients of Class IV had higher average TNF-*α* than patients of Class III and Class II.

All patients underwent biopsy before TNF-*α* test, and the biopsy-test interval was 28 (7-85) months. According to the ISKD classification, most patients were Class II-IV, with 15.1%, 73.6%, and 7.5%, respectively. Class IV patients had significantly higher average TNF-*α* levels than Class II and III patients (Figure 2, *P* = 0.027 for Class II and *P* = 0.009 for Class III).

### 3.4. Comparison of IgAV-N and IgAN

We compared IgAV-N with IgAN patients matched for age, sex, and eGFR grade. The serum TNF-*α* level was comparable in the two groups (Table 3). Other clinical features in the two groups were not statistically different.

## 4. Discussion

TNF-*α* is an important inflammatory factor and has been studied in pediatric IgAV-N patients [9–11, 16, 17]. Zhu et al. found that TNF-*α* was increased in IgAV patients, and IgAV-N patients had the highest level of TNF-*α* compared with patients without kidney involvement. The level of TNF-*α* was also positively correlated with the degree of ISKD pathological grade [9]. Yuan et al. also reported a higher serum TNF-*α* level in IgAV children compared with the healthy group, and the level was higher in patients with renal function impairment than in those without renal function impairment. In addition, the TNF-*α* level in the acute nephritis group was higher than that that in those with chronic nephritis and nephritic syndrome [10]. Ha reported that serum TNF-*α* was significantly higher in proteinuric IgAV children in the acute phase compared with those without renal involvement and those with hematuric IgAV. TNF-*α* levels declined in the convalescent phase compared with the acute phase in those with proteinuric IgAV using matched serum samples from the same patients [11]. These results suggested that in IgAV patients, renal involvement led to a more active inflammatory response, and TNF-*α* was a biomarker of disease severity. However, some studies have shown that serum TNF-*α* was comparable in patients with IgAV-N and those without renal involvement [16, 17]. Heterogeneity of disease severity in the included patients in different studies may be the main reason for the inconsistent results.

There are few studies on serum TNF-*α* levels in adult patients with IgAV-N. Berthelot et al. reported similar TNF-*α* serum concentrations in IgAV patients and controls. However, in their IgAV-N group, patients were older with more hypertension and diabetes, and not all patients with renal impairment underwent renal biopsy [18]. In the present study, a detailed cross-sectional analysis of the relationship between TNF-*α* and the clinicopathologic manifestations in biopsy-proven IgAV-N was carried out. It was found that serum TNF-*α* was significantly higher in the IgAV-N group than in healthy controls and was correlated with renal function and ISKD pathological classification. Albumin was also negatively correlated with serum TNF-*α*, although the relationship between TNF-*α* and urine protein excretion was not significant. This may be because lower albumin indicated inflammatory status. As serum TNF-*α* was correlated with renal function and histopathology, we suspect that TNF-*α* might be a biomarker of renal prognosis in IgAV-N, which we will investigate in future research.

IgAV is frequently reported to follow respiratory tract infections which can activate the immune system and induce IgA production. As the deposition of IgA complexes is a characteristic of IgAV-N, the IgA mucosal immune system may be a key element in the pathogenesis of IgAV-N [2]. Downstream complement activation, inflammatory cell infiltration, and cytokine release also contribute to the disease. TNF-*α*, secreted mainly by monocytes/macrophages, is a proinflammatory cytokine that plays a critical role in mucosal functions including impacting paracellular permeability, wound healing and guiding immune cell attraction, and function [4, 5]. Abnormal TNF-*α* was also proved to cause direct renal injury. Raised serum TNF-*α* concentration may damage vascular endothelium [6, 19] and promote proliferation of glomerular mesangial cells [20]. Genetic research also provides evidence of the role of TNF-*α* in IgAV. Ding et al. reported that the A/A genotype frequency at the +308G/A position of TNF-*α* in IgAV patients was higher than that in surgical patients without renal diseases or rheumatic immune diseases, which indicated that A/A homozygosity of TNF-*α* may result in a genetic predisposition to IgAV [21]. Zhu et al. treated IgAV patients with gastrointestinal involvement with hemoperfusion. Compared with the conventional therapy group, the hemoperfusion group had reduced TNF-*α* levels and other factors such as interleukin 6 and malondialdehyde to a greater extent. The hemoperfusion group reduced the glucocorticoid dosage and the rate of renal involvement in children with severe IgAV, which may benefit from the removal of inflammatory factors [9].

In the present study, we compared serum TNF-*α* in IgAV-N and IgAN patients as these two diseases share similar renal clinicopathologic manifestations and pathogenesis. Serum TNF-*α* levels were comparable in our study. Sugiyama et al. reported significantly higher TNF-*α* in IgAV-N than in IgAN patients [22]. However, the two groups also received a significantly different intensity of treatment which indicated that disease severity of the enrolled patients in the two groups was not comparable. In our previous work, serum TNF-*α* was significantly correlated with eGFR, urine protein excretion, mesangial hypercellularity, and tubular atrophy according to the Oxford classification [12]. Our pilot study showed that hydroxychloroquine, which targets TNF-*α*, was effective in ameliorating proteinuria in selected patients with IgAN [23]. These reports support that TNF-*α* plays a role in IgAN and is a useful biomarker of disease severity. Thus, it is reasonable that serum TNF-*α* was correlated with renal function in IgAV-N and comparable in these two diseases. We also suspect that hydroxychloroquine may be effective in IgAV-N, which requires a further study.

Our research had some limitations. First, it was a cross-sectional design and only provided a correlation analysis. How TNF-*α* can impact or predict renal prognosis requires further research. Second, this was a single center study, and the enrolled patients had relatively mild to moderate disease with well-preserved renal function. Therefore, caution should be used when extending these conclusions to more severe patients. Third, as this was a retrospective study, the biopsy data was obtained before TNF-*α* test with a variable range, which may have had an impact on the relationship between the pathological manifestations and TNF-*α* level. A prospective study enrolling more patients and a follow-up period of at least 3 years to confirm the value of serum TNF-*α* in IgAV-N patients are planned.

## 5. Conclusion

Serum TNF-*α* is correlated with renal function and pathological classification in IgAV-N patients. TNF-*α* is a potential biomarker for the assessment of IgAV-N severity.

## Figures and Tables

**Table 1:** Demographic and clinical features of IgAV-N.

	All patients (*n* = 53)	Low group (TNF-*α* ≤ 8.1 pg/mL, *n* = 35)	High group (TNF-*α* > 8.1 pg/mL, *n* = 18)	*P* value
Age (years)	29 (25-37)	30 (25-36)	28 (21-38)	0.288
Female, no. (%)	36 (67.9%)	24 (68.6%)	12 (66.7%)	1.000
Smoking, no. (%)	1 (1.9%)	0 (0.0%)	1 (5.6%)	0.340
Diabetes, no. (%)	1 (1.9%)	1 (2.9%)	0 (0.0%)	1.000
BMI (kg/m^2^)	23.5 (21.0-26.0)	23.4 (21.3-25.8)	24.1 (21.0-26.8)	0.551
Arthralgia, no. (%)	11 (20.8%)	7 (20.0%)	4 (22.2%)	1.000
Gastrointestinal involvement, no. (%)	9 (16.9%)	7 (20.0%)	2 (11.1%)	0.701
MBP (mmHg)	87 (83-96)	87 (83-93)	93 (87-97)	0.090
sCr (*μ*mol/L)	75 (65-94)	72 (63-85)	83 (68-110)	0.049
eGFR (mL/min/1.73 m^2^)	101.1 (76.9-119.2)	101.1 (86.2-118.1)	91.2 (53.0-123.1)	0.388
G1, no. (%)	35 (66.0%)	26 (74.3%)	9 (50.0%)	0.032^#^
G2, no. (%)	12 (22.6%)	8 (22.9%)	4 (22.2%)	
G3, no. (%)	5 (9.4%)	1 (3.1%)	4 (22.2%)	
G4, no. (%)	0 (0.0%)	0 (0.0%)	0 (0.0%)	
G5, no. (%)	1 (1.9%)	0(0.0%)	1 (5.6%)	
Cys-C (mg/dL)	0.90 (0.77-1.26)	0.87 (0.76-1.09)	1.00 (0.84-1.59)	0.036
Urinary red blood cell (/*μ*L)	53 (30-161)	73 (30-171)	38 (28-83)	0.143
uPCR (mg/g Cr)	540 (236-1001)	446 (211-933)	649 (337-2618)	0.155
PER (g/24 h)	0.66 (0.35-1.38)	0.52 (0.28-1.07)	0.96 (0.47-1.73)	0.128
<1 g/24 h, no. (%)	35 (66.0%)	26 (74.3%)	9 (50.0%)	0.170
1-3 g/24 h, no. (%)	13 (24.5%)	7 (20.0%)	6 (33.3%)	
≥3 g/24 h, no. (%)	5 (9.4%)	2 (5.7%)	3 (16.7%)	
IgA (g/L)	2.79 (2.07-3.53)	2.97 (2.33-3.89)	2.39 (1.58-2.67)	0.004
Albumin (g/L)	43 (39-46)	44 (41-46)	41 (35-45)	0.059
hsCRP (mg/L)	0.75 (0.40-1.68)	0.70 (0.41-1.62)	1.09 (0.28-2.68)	0.460
Treatment, no. (%)				
None	11 (20.8%)	8 (22.9%)	3 (16.7%)	0.730
RASI	31 (58.5%)	22 (62.9%)	9 (50.0%)	1.000
Glucocorticoid	21 (39.6%)	10 (28.6%)	11 (61.1%)	0.037
Imunosuppressants	18 (34.0%)	11 (31.4%)	7 (38.9%)	0.760

IgAV-N: immunoglobulin A vasculitis nephritis; BMI: body mass index; MBP mean blood pressure; sCr: serum creatinine; eGFR: estimated glomerular filtration rate, calculated by Chronic Kidney Disease Epidemiology Collaboration (CKD-EPI) equation [12], and graded by Kidney Disease: Improving Global Outcomes classification [13] (eGFR ≥ 90 (G1), 60–89 (G2), 30–59 (G3), 15–29 (G4), and <15 (G5) mL/min/1.73 m^2^); Cys-C: serum cystatin C; uPCR: urinary protein to serum creatinine ratio; PER: urine protein excretion; hsCRP: hypersensitive C-creative protein; RASI: renin-angiotensin system inhibitors. ^#^Because only one patient was at G5, none of the patients was at G4, and only patients of G1~3 were included in statistics.

**Table 2:** Correlation analysis and multivariate regression analysis of TNF-*α*.

Variables	*r* or *Z*	*P* value	Multivariate regression coefficient *β*	*P* value
Age	0.036	0.799		
Sex	-0.108	0.440		
MBP	0.222	0.113		
Albumin	-0.338	0.014	-0.177	0.189
sCr	0.376	0.006		
eGFR	-0.317	0.021	-0.377	0.007
Cys-C	0.449	0.001		
IgA	-0.257	0.066		
PER	0.197	0.162		
Urinary red blood cell	-0.194	0.165		
hsCRP	0.058	0.713		

MBP: mean blood pressure; sCr serum creatinine; eGFR: estimated glomerular filtration rate; Cys-C: serum cystatin C; PER: urine protein excretion; hsCRP: hypersensitive C-creative protein.

**Table 3:** Comparison of IgAV-N and IgAN.

	IgAV-N	IgAN	*P* value
Age (years)	29 (25-37)	29 (27-34)	0.228
Female, no. (%)	36 (67.9%)	35 (66.0%)	0.836
Diabetes, no. (%)	1(1.9%)	1(1.9%)	1.000
MBP (mmHg)	87 (83-96)	87 (78-93)	0.235
TNF-*α* (pg/mL)	7.4 (5.7-9.4)	8.0 (6.7-9.9)	0.116
sCr (*μ*mol/L)	75 (65-94)	79 (69-93)	0.359
eGFR (mL/min/1.73 m^2^)	101.1 (76.9-119.2)	102.8 (80.8-121.9)	0.716
Cys-C (mg/dL)	0.90 (0.77-1.26)	0.93 (0.77-1.11)	0.987
Urinary red blood cell (/*μ*L)	53 (30-161)	39 (9-108)	0.080
uPCR (mg/g Cr)	540 (236-1001)	524 (183-986)	0.897
PER (g/24 h)	0.66 (0.35-1.38)	0.76 (0.44-1.30)	0.663
Albumin (g/L)	43 (39-46)	43 (42-45)	0.512
IgA (g/L)	2.79 (2.07-3.53)	2.38 (1.92-3.22)	0.165
Glucocorticoid, no. (%)	21 (39.6%)	28 (52.8%)	0.173
Immunosuppressants, no. (%)	18 (34.0%)	28 (52.8%)	0.050

IgAV-N: immunoglobulin A vasculitis nephritis; IgAN: immunoglobulin A nephropathy; MBP: mean blood pressure; sCr: serum creatinine; eGFR: estimated glomerular filtration rate; Cys-C: serum cystatin C; uPCR: urinary protein to serum creatinine ratio; PER: urine protein excretion.

## Data Availability

The data used to support the findings of this study are available from the corresponding author upon request.
